# Identification and Validation of a Novel Six-Gene Prognostic Signature of Stem Cell Characteristic in Colon Cancer

**DOI:** 10.3389/fonc.2020.571655

**Published:** 2021-02-19

**Authors:** Yichao Liang, Qi Su, Xin Wu

**Affiliations:** Department of General Surgery, Shengjing Hospital of China Medical University, Shenyang, China

**Keywords:** colon adenocarcinoma, stem cell, prognostic marker, six-gene signature, molecular subtype

## Abstract

Cancer stem cells play crucial roles in the development of colon cancer (COAD). This study tried to explore new markers for predicting the prognosis of colon cancer based on stem cell-related genes. In our study, 424 COAD samples from TCGA were divided into three subtypes based on 412 stem cell-related genes; there were significant differences in prognosis, clinical characteristics, and immune scores between these subtypes. 694 genes were screened between subgroups. Subsequently a six-gene signature (DYDC2, MS4A15, MAGEA1, WNT7A, APOD, and SERPINE1) was established. This model had strong robustness and stable predictive performance in cohorts of different platforms. Taken together, the six-gene signature constructed in this study could be used as a novel prognostic marker for COAD patients.

## Introduction

Colon cancer is one of the most common malignant tumors in the world, with approximately 1.2 million new cases and 600,000 deaths each year (1). The 5-year survival rate of patients with early-stage colon cancer who undergo radical resection is more than 90%, but the lack of specific clinical manifestations makes the early diagnosis of noninvasive tumors difficult. Many patients are diagnosed with advanced colon cancer and metastasis, and the 5-year survival rate drops to around 10% (2). Despite improvements in surgery, chemotherapy, and radiotherapy, the current treatment of colon cancer is still not satisfactory (3). Therefore, exploring the mechanisms of colon cancer development and prognostic markers is needed to help prevent and treat colon cancer.

In recent years, research has shown that there is a small group of special cells inside tumors. These cells have strong self-renewal and tumorigenic abilities and stem cell-like characteristics. Such cells are called cancer stem cells (CSCs). CSCs may be involved in the development, recurrence, metastasis, and chemotherapy resistance of tumors and promote tumor progression (4, 5). CSCs are tumor-originating cells mutated from adult stem cells that have self-renewal and unlimited proliferation capacities, and they can produce different types of tumor cells through self-renewal and differentiation (5, 6). The ability of CSCs to initiate and maintain cancer cell reproduction is a necessary condition for metastasis. Tumor stem cells are heterogeneous and include subpopulations with metastatic capacity (7–9). In addition, CSCs can stay dormant for a long time and have multiple molecular mechanisms of drug resistance, but they are not sensitive to external physical and chemical factors that kill tumor cells (10, 11). Because CSCs play important roles in colon cancer progression, CSC markers or CSC regulatory pathways may become prognostic markers and potential therapeutic targets for patients with colon cancer.

At present, the main colon cancer stem cell markers include CD133, CD44, CD90, ALDH1A1, EpCAM, SOX2, SOX9, LGR5, *etc*., and they are usually used to identify and isolate CSCs (12–16). CD133, CD44, EpCAM, LGR5, ALDH1A1, SOX2, and SOX9 are prognostic markers for bowel cancer, and they have a role in cancer diagnosis and predicting the pathologic stage. SOX2 and SOX9 are transcription factors that maintain the characteristics of embryonic stem cells and participate in the formation of tumors. In addition, the study identified a series of molecules that affect the prognoses of patients by regulating the characteristics of colon cancer stem cells. For example, Cullin-4B (*CUL4B*) upregulates the expression of the CSC marker CD44 to maintain colon cancer stemness and drive malignant progression to affect prognosis (17). In addition, multiple signaling pathways are involved in CSC regulation. For example, the classic Wnt signaling pathway is essential for the maintenance of stem cells. After Wnt binds to its receptor, Frizzled, the Wnt signaling pathway is activated, which maintains the asymmetric division, stable number, and specific differentiation function of stem cells, thereby regulating the tumor cell Dryness affects the prognoses of patients (18–20). However, research is currently limited, so it is difficult to systematically study the relationships between colon cancer stem cell markers and related pathways and patient prognoses.

In this study, publicly available data were used to develop and validate a six-gene signature prognostic stratification system based on colon cancer stem cells. The model had a satisfactory area under the curve (AUC) in both training and validation cohorts, and it was independent of clinical characteristics. Therefore, it was recommended to use this classifier as a molecular diagnostic test to assess the prognostic risk of patients with colon cancer.

## Material and Methods

### Source of Cancer Stem Cell-Related Genes

The human CSC-related pathways were downloaded from the Molecular Signature Database v7.0 (MSigDB), and 456 genes related to CSCs were sorted from the 30 lipid metabolism pathways in **Table 1** **(** **S1_Table**).

**Table 1:** Pathways related to cancer stem cells in reactome and GO databases.

Stem cell function related pathways	Pathway ID	Gene Count
GO : Somatic Stem Cell Population Maintenance	GO:0035019	72
GO : Negative Regulation of Stem Cell Differentiation	GO:2000737	20
GO : Stem Cell Proliferation	GO:0072089	118
GO : Hematopoietic Stem Cell Differentiation	GO:0060218	79
GO : Negative Regulation of Stem Cell Proliferation	GO:2000647	16
GO : Stem Cell Division	GO:0017145	41
GO : Hematopoietic Stem Cell Proliferation	GO:0071425	23
GO : Positive Regulation of Stem Cell Differentiation	GO:2000738	20
GO : Regulation of Stem Cell Population Maintenance	GO:2000036	28
GO : Neuronal Stem Cell Population Maintenance	GO:0097150	22
GO : Regulation of Stem Cell Proliferation	GO:0072091	67
GO : Somatic Stem Cell Division	GO:0048103	24
GO : Stem Cell Differentiation	GO:0048863	248
GO : Positive Regulation of Stem Cell Proliferation	GO:2000648	40
GO : Regulation of Stem Cell Differentiation	GO:2000736	112
GO : Hematopoietic Stem Cell Migration	GO:0035701	6
GO : Stem Cell Fate Commitment	GO:0048865	9
GO : Mesenchymal Stem Cell Maintenance Involved In Nephron Morphogenesis	GO:0072038	6
GO : Mesenchymal Stem Cell Differentiation	GO:0072497	8
GO : Mesenchymal Stem Cell Proliferation	GO:0097168	5
GO : Asymmetric Stem Cell Division	GO:0098722	10
GO : Regulation of Hematopoietic Stem Cell Proliferation	GO:1902033	9
GO : Positive Regulation of Hematopoietic Stem Cell Proliferation	GO:1902035	5
GO : Negative Regulation of Stem Cell Population Maintenance	GO:1902455	8
GO : Positive Regulation of Stem Cell Population Maintenance	GO:1902459	8
GO : Regulation of Somatic Stem Cell Population Maintenance	GO:1904672	7
GO : Negative Regulation of Somatic Stem Cell Population Maintenance	GO:1904673	5
GO : Regulation of Stem Cell Division	GO:2000035	10
GO : Regulation of Mesenchymal Stem Cell Differentiation	GO:2000739	6
Reactome Transcriptional Regulation of Pluripotent Stem Cells	R-HSA-452723	31

### Data Collection and Downloading

The Cancer Genome Atlas (TCGA) Genomic Data Commons (GDC) application performing interface (API) was used to download the latest expression data and clinical follow-up information of patients with colon adenocarcinoma (COAD). This cohort contained RNA sequencing data and clinical follow-up information of 424 samples (**S2_Table**).

The GSE39582 chip expression data in MINiML format were downloaded from the National Center for Biotechnology Information (NCBI). GSE39582 contained 536 samples with clinical characteristics (**S3_Table**), and the gene expression profile is shown in **S4_Table**. GSE17536 contained 144 samples with clinical characteristics (**S5_Table**), and the gene expression profile is shown in **S6_Table**. These three cohorts were included because they were the largest sample sets in the same platform with detailed follow-up information of colorectal cancer.

### Data Preprocessing

The following steps were performed to preprocess the RNA sequencing data from the TCGA samples. 1) Remove the samples without clinical information or with progression free survival (PFS) <30 days. 2) Remove the data of the normal tissue samples. 3) Remove any gene whose fragments per kilobase million (FPKM) is 0 in half the samples. 4) Retain the CSC-related gene expression profiles.

The following steps were conducted to preprocess the GSE39582 and GSE17536 cohorts. 1) Remove the data of the normal tissue samples. 2) Convert the disease-free survival (DFS) data by year or month into by day. 3) Remove the samples with DFS <30 days. 4) Use the “Bioconductor” package in R to map the chip probe to the human gene SYMBOL. 5) Retain the CSC-related gene expression profiles.

We selected primary cancer samples, all of which were from before the first treatment, and we excluded samples whose follow-up time was <30 days. In addition, we randomly divided the TCGA cohort into 2 groups, one as the training set and the other as the internal validation set. Two Gene Expression Omnibus (GEO) cohorts were used as independent external verification sets. Since we wanted to verify the predictive performance of the model in different platforms, we did not specially process the GEO expression profile datasets.

The statistical information of the preprocessed cohorts is shown in **Table 2**.

**Table 2:** Clinical information of the three pre-processed cohorts.

Characteristic		Training Set (n = 318)	Entire Set (n = 424)	p value	GSE39582 (n = 536)	GSE17536 (n = 144)
**Age (years)**	≤60	96	131	0.899	143	46
	>60	222	293		393	98
**Survival Status**	Living	237	310	0.727	389	108
	Dead	81	114		147	36
**Gender**	female	145	195	0.974	240	69
	male	173	229		296	75
**pathologic_T**	T1	6	10	0.907	12	–
	T2	59	73		47	–
	T3	221	293		349	–
	T4	32	47		105	–
**pathologic_N**	N0	184	248	0.905	293	–
	N1	80	101		127	
	N2/N3	54	75		92	–
**pathologic_M**	M0	238	314	0.857	485	–
	M 1/M X	76	105		32	–
**Tumor Stage**	Stage I	53	70	0.994	37	24
	Stage II	119	162		260	55
	Stage III	91	122		205	55
	Stage IV	46	59		30	10
**Lymphatic invasion**	Yes	118	147	0.642	–	–
	No	173	235		–	–
**Venous invasion**	Yes	67	88	1	–	–
	No	214	279		–	–

### Identification of Molecular Subtypes by the Non-Negative Matrix Factorization Algorithm

First, the expression profile data of the CSC-related genes were extracted from the TCGA database. Genes with expression >0 in more than half the samples were retained. A total of 412 genes were included in subsequent analysis. COAD samples were clustered by non-negative matrix factorization (NMF) with the standard of “brunet” and 50 iterations. The number of clusters, k, was 2 to 10. The average contour width of the common member matrix was determined through “NMF” in the R package, and the minimum number of member of each subclass was 10. The optimal number of clusters was determined based on cophenetic, dispersion, silhouette, and so on.

### Analysis of Differentially Expressed Genes Between Subtypes

DESeq2 was used to calculate the differentially expressed genes (DEGs) between the subtypes. With a false discovery rate (FDR) <0.05 and |log2FC| >1 as the threshold, functional enrichment analysis of the DEGs was conducted using the R software package “WebGestalt” to explore the pathways and functions involved with these DEGs.

### Risk Model Construction in the Training Cohort

First, 75% of the 424 TCGA samples after preprocessing were randomly selected as the training cohort. To avoid random allocation bias affecting the stability of subsequent modeling, 100 repeated samplings with replacements for all samples were performed in advance to ensure that the distribution of the randomly selected samples was consistent with all samples in terms of age, clinical stage, and tumor-node-metastasis (TNM) stage. For all DEGs and survival data, univariate Cox proportional hazards regression analysis was performed. We then used the R package survival “coxph” function, with log rank p <0.01 as the threshold. It was necessary to further reduce the gene range and construct a prognostic model while maintaining a high accuracy rate, and the least absolute shrinkage and selection operator (Lasso) method was used as a compression estimate. It constructed a penalty function to obtain a more refined model so that it compressed some coefficients, and at the same time it set some coefficients to 0. Therefore, the advantage of subset shrinkage was preserved. It was a kind of processing biased estimation with complex collinearity data, which could realize variable selection at the same time as parameter estimation and better solve the multicollinearity problem in regression analysis.

### Univariate and Multivariate Analyses

In the clinical information of the TCGA data, the related hazard ratios (HRs), 95% confidence intervals (CIs) of the HRs, and p-values were analyzed using univariate and multivariate Cox regression. Age, sex, pathologic T, N, and M stage, and tumor stage information was systematically analyzed to evaluate the clinical independence and predictive performance of our model.

### Risk Scores and Potentially Relevant Regulatory Pathways

To observe the relationships between the risk scores of the different samples and biological functions, the gene expression profiles corresponding to these samples were selected for single-sample gene set enrichment analysis (ssGSEA) using the R software package “GSVA,” and the score of each sample for different functions was calculated to obtain the correspondence of each function. The ssGSEA score of each sample was used to further calculate the correlations between functions and risk scores.

### Quantitative PCR

Sixty cases, with matched non-tumorous and tumorous tissue samples from Shengjing Hospital of China Medical University, were enrolled in this study. Total RNA was extracted using TRIzol reagent (Invitrogen, Carlsbad, CA, USA) following the manufacturer’s instructions. Complementary DNA (cDNA) was synthesized from high quality total RNA using PrimeScript™ RT Master Mix (No. RR036A, Takara Bio USA, Mountain View, CA, USA). Real-time qPCR was performed to validate gene expression using Power SYBR™ Green PCR Master Mix (No. A25742, Thermo Fisher Scientific, Waltham, MA, USA) on the 7900HT Fast Real-Time PCR System (Applied Biosystems, Foster City, CA, USA). Relative expression was calculated based on 2-ΔΔCt method.

## Results

### Molecular Subtype Identification

**Figure 1:**
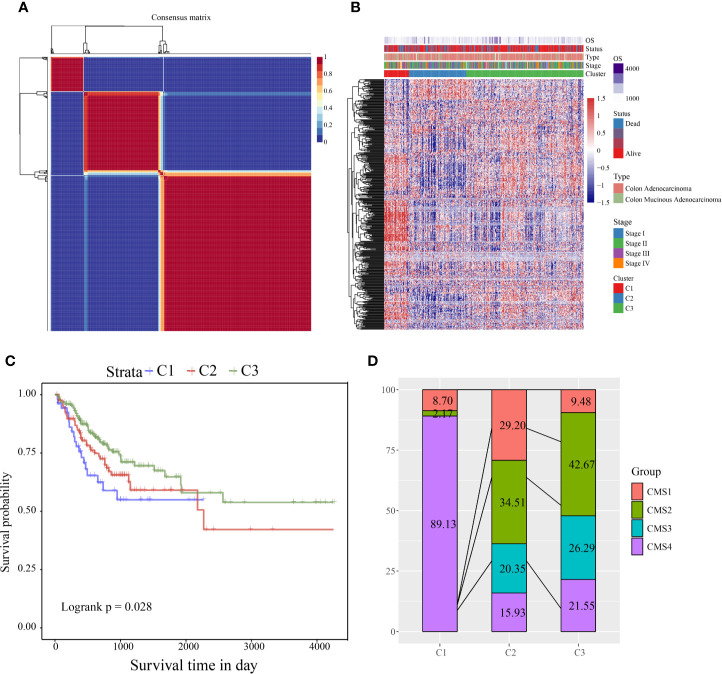
**(A)** Consensus map of NMF clustering; **(B)** heat map of clustering of 412 prognosis-related genes **(C)** PFS prognostic survival curve of different molecular subtypes; **(D)** comparison of different subtypes and CMS, where different colors represent different subtypes. The coordinates represent the percentage of samples.

According to the cophenetic, dispersion, and silhouette indicators, the optimal number of clusters was determined to be three (**Figure 1A**, **Figure S1**). **Figure 1B** shows the expression of the CSC-related genes related to prognosis in the two subcategories. The gene expression in C1 was higher than in C2 and C3. Further, we analyzed the prognostic relationship between the three groups, and the results showed that C1 had the worst prognosis, C3 had the best prognosis, and there was a significant difference between the three (log rank p = 0.028; **Figure 1C**). Further analysis revealed a significant difference between C1 and C3 (log rank p = 0.29; **Figure S1A**). There was no significant difference between C1 and C2 (log rank p = 0.014; **Figure S1B**), and C2 and C3 showed a significant margin (log rank p = 0.089; **Figure S1C**). We also compared the published consensus molecular subtypes of colon cancer and found that the C1 and CMS4 subtypes were highly similar (89.13%), as shown in **Figure 1D**.

### Comparison of Clinical Features Between Molecular Subtypes

According to the CSC-related gene sets, COAD samples were clustered into three subcategories, and the clinical features (clinical stage; T, N, and M stages; sex; and age) among the three subtypes were compared. Age, M stage showed significant differences in the three groups (**Figure S2**). Furthermore, using the TIMER tool, the immune scores of the three subtypes were compared. We found that the immune scores of the C1 subtype for CD4 T cells, CD8 T cells, neutrophils, macrophages, and dendritic cells were significantly higher than those of the C2 and C3 subtypes (**Figure S3B–F**). For B cells, the immune score of the C1 subtype was significantly higher than that of the C2 subtype, but the relationship with the C3 subtype was not significant (**Figure S3A**). This might indicate that the immune invasion of COAD has a complicated relationship with prognosis and stem cells. For all immune cell scores in all samples, see **S7_Table**.

### Identification of Differentially Expressed Genes

DESeq2 was used to calculate the DEGs between the C1 and C2 and the C1 and C3 subtypes. After filtering according to the threshold FDR <0.05 and |log2FC| >1, 506 were C1/C2 and 463 were C1/C3. A volcano map of the DEGs between C1 and C2 is shown in **Figure S4A**. There was mainly upregulated differential expression between C1 and C2. A volcano map of the DEGs between C1 and C3 is shown in **Figure S4B**. The DEGs are shown in **S8_Table**. We analyzed the intersections of the genes with differences between subtypes (**Figure S4C**), and there was much intersection of the DEGs between subtypes.

### Functional Analysis of Differentially Expressed Genes

We conducted functional enrichment analysis of the 694 DEGs through the R software package “WebGestalt,” and we selected the threshold FDR <0.05 (**S9_Table**). There were 14 enriched pathways, including the extracellular matrix (ECM)-receptor interaction pathway, the PI3K-Akt signaling pathway, and other pathways related to tumor development (**Figure S5A**). There were 43 pathways enriched in the Reactome database, of which the main ones were ECM proteoglycans, the MET-activated PTK2 signaling pathway, and other pathways (**Figure S5B**). Gene Ontology (GO) enrichment results showed 100 cellular components (CCs) and 74 molecular functions (MFs). The top six CC and MF terms are shown in **Figures S5C, 5D**. They were mainly related to the ECM, receptor ligand activity, receptor regulator activity, and other receptor regulator molecular functions.

### Risk Model Construction in the Training Cohort

**Figure 2:**
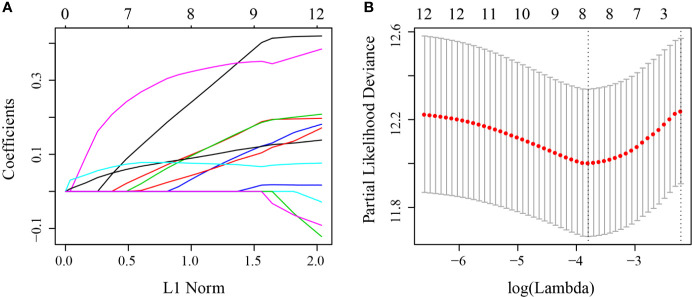
**(A)** Change trajectory of each independent variable; the horizontal axis represents the log value of the independent variable lambda, and the vertical axis represents the coefficient of the independent variable; **(B)** the confidence interval at different value of lambda.

First, 75% of the 424 TCGA samples after preprocessing were randomly selected as the training cohort to construct the model. The sample information of the final training cohort is shown in **Table 2**. For the DEG and survival data, univariate Cox proportional hazards regression analysis was performed. Using the R package survival “coxph” function, log rank p <0.01 was selected as the threshold, and 14 genes with significant prognostic differences were identified (**S10_Table**). Using the R software package “glmnet” for Lasso-Cox regression analysis, the change trajectory of each independent variable was first analyzed (**Figure 2A**). As the lambda gradually increased, the number of independent variable coefficients tending to 0 also gradually increased. A 10-fold cross-validation was used for the model construction, and the CI under each lambda was analyzed (**Figure 2B**). The model was optimal when lambda = 0.02241157. Eight genes with lambda = 0.02241157 were selected as the target genes. Further, we conducted the multivariate Cox survival analysis on the 8 genes obtained in the previous step, and we retained the six mRNAs with the smallest Akaike information criterion (AIC) value (AIC = 810.96) as the final model. The details of the six mRNAs are shown in **Table 3**. The prognostic Kaplan–Meier (KM) curves of these six genes are shown in **Figure S6**. Four genes (*MAGEA1*, *WNT7A*, *APOD*, and *SERPINE1*) could significantly classify the TCGA training cohort into high- and low-risk groups (p <0.05), while *DYDC2* and MS4A15 could not significantly do this. The formula of the final six-gene signature was as follows: RiskScore_6_ = 0.4448*exp^DYDC2^ + 0.21*exp^MS4A15^ + 0.2098*exp^MAGEA1^ + 0.5068*exp^WNT7A^ + 0.1363*exp^APOD^ + 0.1545*exp^SERPINE1^.

**Table 3:** Information of the six-mRNA signature.

Symbol	coef	HR	Z-score	P value	Low 95%CI	High 95%CI
DYDC2	0.445	1.56	2.21	0.027	1.051	2.316
MS4A15	0.210	1.23	1.77	0.077	0.977	1.557
MAGEA1	0.210	1.23	2.56	0.010	1.051	1.448
WNT7A	0.507	1.66	2.58	0.010	1.130	2.438
APOD	0.136	1.15	1.97	0.049	1.0001	1.312
SERPINE1	0.154	1.17	1.89	0.059	0.994	1.370

### Receiver Operating Characteristic Analysis of the Risk Model

**Figure 3:**
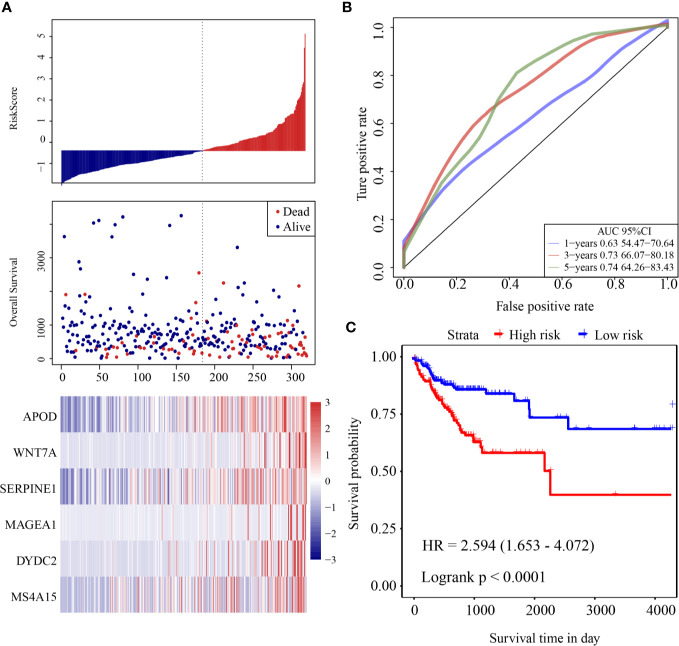
**(A)** Risk score, survival time and survival status and expression of six-gene in the training cohort; **(B)** ROC curve and AUC of six-gene signature classification; **(C)** KM survival curve distribution of six-gene signature in the training cohort.

The risk score of each sample was calculated according to expression. The risk score distribution is shown in **Figure 3A**. Samples with high risk scores had significantly lower overall survival (OS) than those with low risk scores, suggesting that the samples with high risk scores had worse prognoses. High expression of six genes (*DYDC2*, *MS4A15*, *MAGEA1*, *WNT7A*, *APOD*, and *SERPINE1*) was associated with high risk, so these genes were considered risk factors. Receiver operating characteristic (ROC) analysis of the prognostic classification of the risk score was performed using the R software package “timeROC.” The prognostic classification efficiency at 1-year, 3-years, and 5-years was analyzed (**Figure 3B**). The 5-year AUC of the model was 0.74. The KM curve is shown in **Figure 3C**. There was an extremely significant difference between the high- and low-risk groups (log rank p < 0.0001, HR = 2.594).

### Validation of the Robustness of the Six-Gene Signature in the Internal Cohort

To evaluate the robustness of the model, the same model and coefficients as those in the training cohort were used in the validation cohort and all cohorts from TCGA. The risk score of each sample was calculated according to expression.

**Figure 4:**
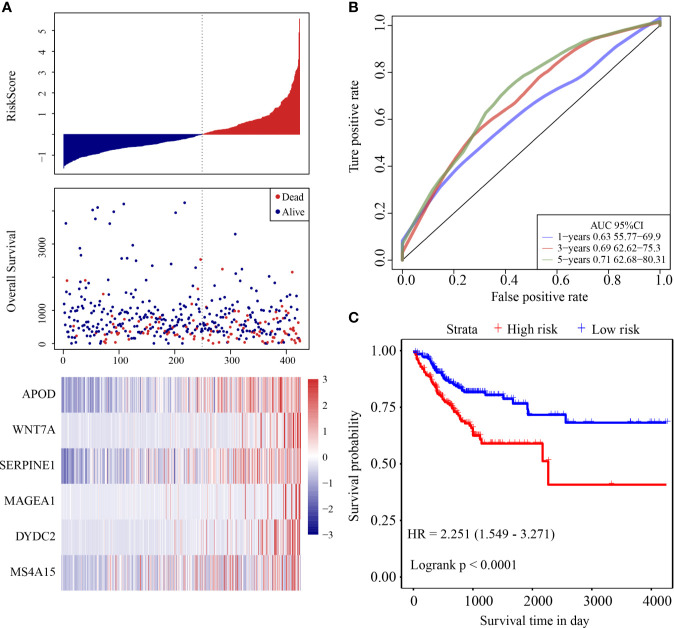
**(A)** Risk score, survival time and survival status and expression of the six genes in the internal validation cohort; **(B)** ROC curve and AUC of the six-gene signature classification; **(C)** KM survival curve distribution of six-gene signature in validation cohort.

The risk score distribution of the validation cohort is shown in **Figure 4A**. Samples with high risk scores had significantly lower OS than those with low risk scores, suggesting that samples with high risk scores had worse prognoses. High expression of six genes (*DYDC2*, *MS4A15*, *MAGEA1*, *WNT7A*, *APOD*, and *SERPINE1*) was associated with high risk, so these genes were considered risk factors. ROC analysis of the prognostic classification of the risk score was performed using the R software package “timeROC.” The prognostic classification efficiency at 1-year, 3-years, and 5-years was analyzed (**Figure 4B**). The 5-year AUC of the model was 0.71. The KM curve is shown in **Figure 4C**. There was an extremely significant difference between the high- and low-risk groups (log rank p < 0.0001, HR = 2.251).

### Validation of the Robustness of the Six-Gene Signature in the External Cohorts

The same model and coefficients as those in the training cohort were used in the two external validation cohorts. The risk score of each sample was calculated according to expression, and the risk score distributions of the two validation cohorts were drawn.

**Figure 5:**
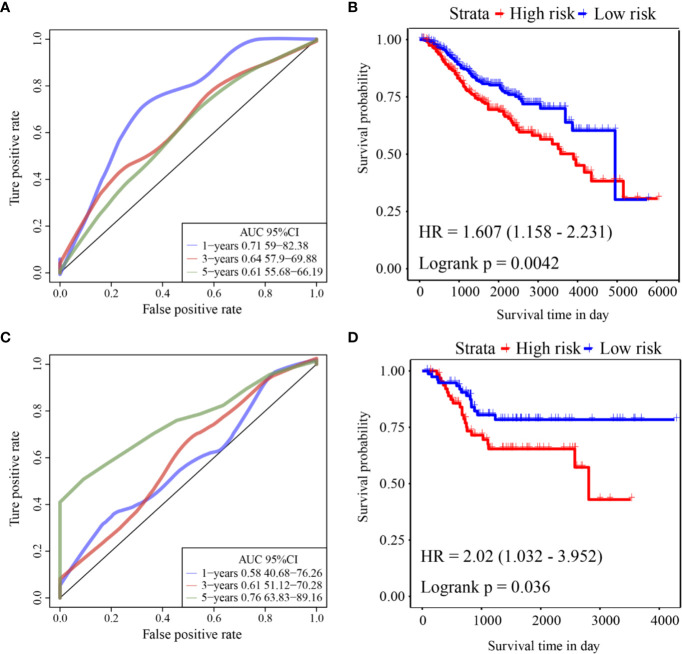
**(A)** ROC curve of GSE39582 external validation cohort; **(B)** KM curve of the six-gene signature in GSE39582 external validation cohort; **(C)** ROC curve of GSE17536 external validation cohort; **(D)** KM curve of the six-gene signature in GSE17536 external validation cohort.

ROC analysis of the prognostic classification of the risk score in GSE39582 was performed using the R software package “timeROC” to analyze the prognostic classification efficiency at 1-year, 3-year, and 5-years. The ROC curves of this model are shown in **Figure 5A**. The AUC for 1-year was 0.71. The KM curve is shown in **Figure 5B**. There was a significant difference between the high- and low-risk groups (log rank p = 0.0042, HR = 1.607).

ROC analysis of the prognostic classification of the risk score in GSE17536 was also performed using “timeROC” to analyze the prognostic classification efficiency at 1-year, 3-years, and 5-years. The ROC curves of this model are shown in **Figure 5C**. The AUC for 5-years was as high as 0.76. The KM curve is shown in **Figure 5D**. There was a significant difference between the high- and low-risk groups (log rank p = 0.036, HR = 2.02).

### Risk Model and Prognostic Analysis of Clinical Characteristics

**Figure 6:**
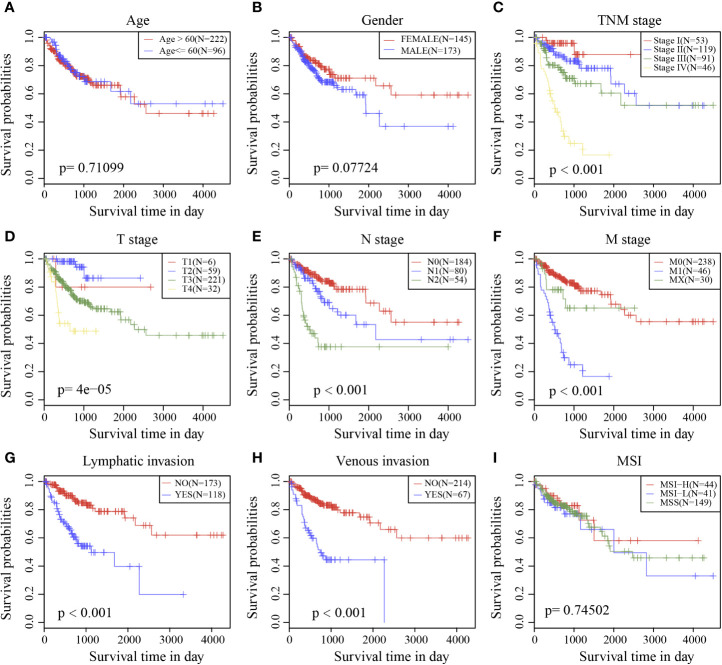
Prognostic survival curves of different clinical characteristics. **(A)** age; **(B)** gender; **(C)** Clinical Stage; **(D)** T stage; **(E)** N stage; **(F)** M stage; **(G)** Lymphatic invasion; **(H)** Venous invasion; **(I)** MSI; The abscissa represents survival time, and the ordinate represents Survival rate.

Survival analysis showed that clinical stage, T stage, N stage, M stage, lymphatic invasion, and venous invasion in the TCGA training cohort samples were significantly correlated with PFS in COAD (**Figures 6C–H**). However, age, sex, and MSI had no significant relationship with PFS (**Figures 6A, B, I** **)**.

### Univariate and Multivariate Analyses of the Six-mRNA Signature

**Figure 7:**
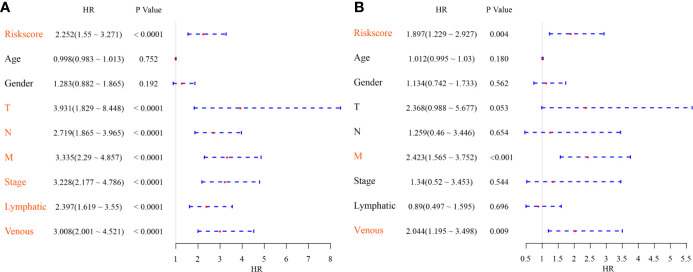
**(A)** Forest map of univariate survival analysis; **(B)** Forest map of multivariate survival analysis, where orange red represents significant PFS correlation.

To identify the independence of the Six-mRNA signature model in clinical application, the relevant HRs, 95% CIs of the HRs, and p-values were calculated by univariate and multivariate Cox regression analyses using the clinical information of the TCGA cohort. The clinical information, including age, sex, T stage, N stage, M stage, clinical stage, and Riskscore, was systematically analyzed. Univariate Cox regression analysis showed that clinical factors such as risk score, T stage, N stage, M stage, clinical stage, lymphatic invasion, and venous invasion were significantly related to PFS (**Figure 7A**), but the corresponding multivariate Cox regression analysis showed that only risk score (HR = 1.897, 95% CI = 1.229–2.927, log rank p = 0.004), M stage, and venous invasion were independent prognostic risk factors (**Figure 7B**). This indicated that the six-mRNA signature model had good predictive performance in clinical application.

### Risk Scores and Potentially Related Regulatory Pathways

**Figure 8:**
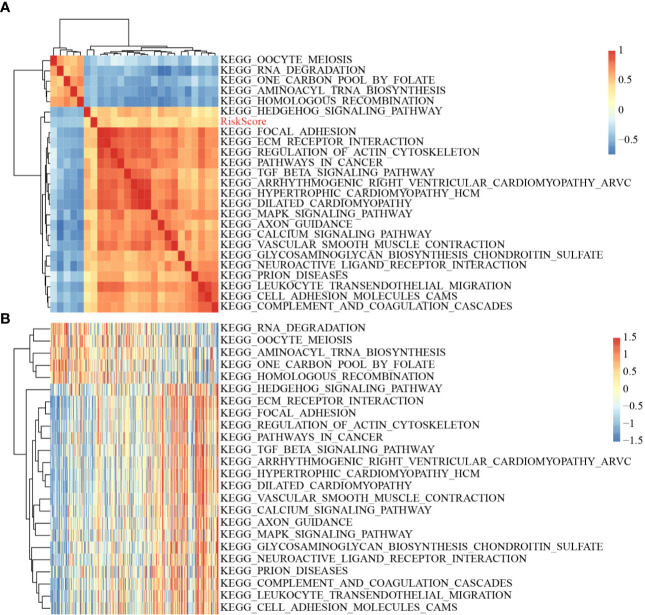
**(A)** Clustering of correlation coefficients between KEGG pathways with correlation to Risk score greater than 0.35 and between Risk scores; **(B)** Changes in ssGSEA scores of KEGG pathways with correlation to risk score greater than 0.35 in each sample, the horizontal axis represents the samples, and the risk scores increase from left to right.

The scores of all samples in terms of the different functions were calculated to obtain the ssGSEA scores then the correlations between these functions and risk scores were further assessed. The functions with correlations >0.35 are shown in **Figure 8A**; most of the functions had a positive correlation with risk score, while the others had a negative correlation. There were 24 Kyoto Encyclopedia of Genes and Genomes (KEGG) pathways with larger correlations, and these were selected to conduct cluster analysis based on enrichment scores (**Figure 8B**). Of these 24 pathways, the *activity of the* ECM–receptor interaction pathway, the TGF beta signaling pathway, *etc.* increased as the risk score rose, while the activity Hedgehog signaling pathway, RNA degradation pathway, *etc.* decreased as the risk score rose, which suggested that the imbalance of these pathways was closely related to tumor development.

### Comparison of the Risk Model With Other Models

**Figure 9:**
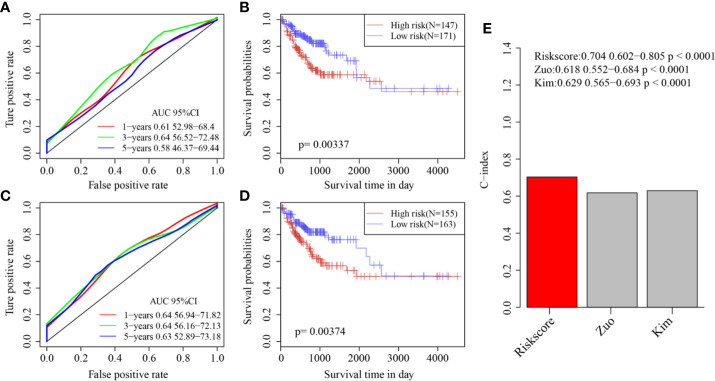
**(A)** AUC curve of Zuo model in TCGA training cohort; **(B)** KM curve of Zuo model in TCGA training cohort; **(C)** AUC curve of Kim model in TCGA training cohort; **(D)** KM curve of Kim model in TCGA training cohort. **(E)** C-index score of three models.

Finally, two prognostic risk models were selected—a six-gene signature (Zuo) and an 11-gene signature (Kim)—for comparison with our six-gene model. To make the models comparable, the same method was used to calculate the risk scores of each COAD sample in the TCGA cohort to evaluate the ROC of each model, and divide the sample into Risk-H and Risk-L groups according to the median risk score to calculate the prognostic differences between the two groups of samples. The ROC and KM curves of the two models are shown in **Figures 9A–D**. The 5-year AUC of the Zuo model and Kim model was 0.58 and 0.63, respectively; these AUCs were worse than that of our six-gene model at 5 years (0.74). The prognoses of the Risk-H and Risk-L samples of these two models also differed significantly (log rank p <0.05). To compare the predictive performance of these models in COAD samples, the “rms” package in R was used to calculate the C-indexes (concordance indexes) of the three models. The C-index of our six-gene model was the highest (above 0.7; **Figure 9**).

### Expression Levels and Prognosis Value of Six Genes

**Figure 10:**
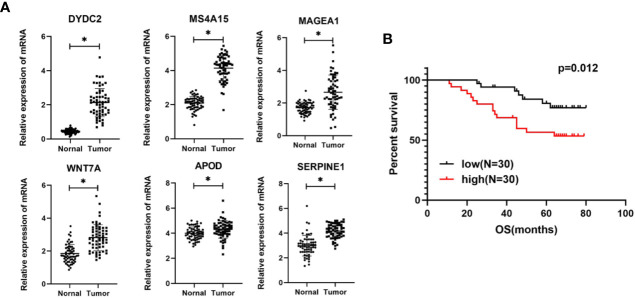
**(A)** Expression levels of six genes quantified using qPCR in 60 paired normal tissues and colon cancer tissues. *P < 0.05. **(B)** Kaplan–Meier curves of OS in COAD patients based on risk score.

Based on the bioinformatics analysis results, the expression of six genes were verified in 60 colon cancer tissues and paired normal tissues. The results in **Figure 10A** showed that the mRNA expression of DYDC2, MS4A15, MAGEA1, WNT7A, APOD, and SERPINE1 were increased in colon cancer tissues (p < 0.05). It was consistent with that analyzed using bioinformatic analysis. Furthermore, the risk score of each COAD patient was calculated using the same formula. Patients were also divided into a high-risk group and a low-risk groups (N = 30 and 30, respectively) by the risk score. A significant difference in OS was found between the high-risk group and low-risk group (**Figure 10B**, P = 0.012).

### Flowchart

**Figure 11:**
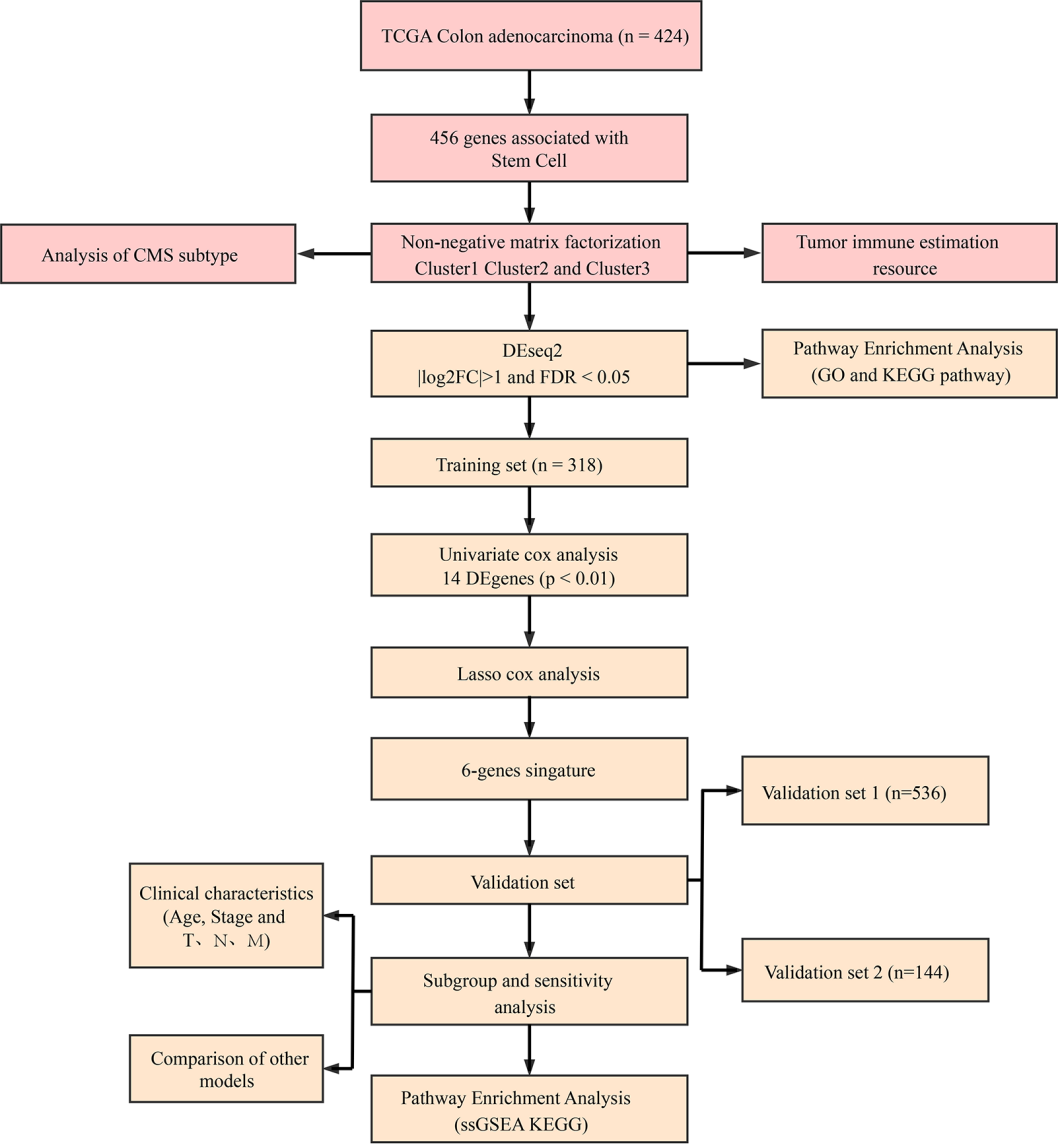
Methodology flow chart.

To make this article easier to understand, we drew a flowchart (**Figure 11**).

## Discussion

Colon cancer is a common malignant tumor of the digestive tract that can occur anywhere from the cecum to the rectum. It is the third most common malignancy worldwide (21). Despite many advances in comprehensive treatment strategies for colon cancer, effective prognostic markers and molecular targeted therapies are still lacking (22, 23). CSCs are a group of heterogeneous cells with different differentiation states. Very few cancer cell subpopulations in tumor tissues have stem cell properties. However, their differentiation potential and unlimited proliferation and self-renewal abilities are decisive factors for tumorigenesis, tumor development, invasion, metastasis, recurrence, and drug resistance. In this study, we identified three molecular subtypes of colon cancer (C1, C2, and C3) based on stem cell-related genes using the NMF algorithm. Prognoses varied significantly between the subtypes and were related to clinical pathologic parameters and immune scores. Using the DESeq2 algorithm, 694 DEGs between each subtype were identified. These genes were enriched in the ECM-receptor interaction pathway and other pathways closely related to cancer development. Finally, a six-gene signature was constructed by the Lasso method and multivariate Cox analysis. The six-gene signature had stable and consistent predictive performance in the TCGA internal and external validation cohorts, and it was significantly related to patients’ clinical and pathologic characteristics and matrix scores. More importantly, it showed independent predictive ability for prognosis in different cohorts. As the six-gene signature showed stable and consistent predictive performance in terms of the prognoses of patients of different platforms, it has great potential in clinical practice.

In the six-gene signature screened and verified based on CSC-related genes, *DYDC2*, *MS4A15*, *MAGEA1*, *WNT7A*, *APOD*, and *SERPINE1* were all risk factors. The protein encoded by *MS4A15* belongs to the membrane-spanning four-domain family, subfamily A (MS4A). MS4A family genes are abnormally expressed in a variety of solid tumors (24–28). In addition, low expression of MS4A family genes is associated with poor prognosis of diffuse gastric cancer (29). These results show that although the mechanism is not clear, *MS4A15* could still be a potential prognostic marker in tumors. Melanoma-associated antigen 1 (MAGEA1) is a member of the melanoma-associated antigens family A (MAGE-A) and is closely related to the prognoses of various malignant tumors, such as esophageal squamous cell carcinoma (30), lung cancer (31), gastric cancer (32), liver cancer (33), and breast cancer (34). The protein encoded by *WNT7A* is an important member of the Wnt protein family and is essential for the activity of the Wnt pathway. *WNT7A* is closely related to the prognoses of multiple solid tumors, such as pancreatic cancer (35), oral squamous cell carcinoma (36, 37), and lung cancer (38). Apolipoprotein D (APOD) is associated with the prognoses of prostate cancer (39) and breast cancer (40). The protein encoded by *SERPINE1* belongs to the family of serine protein kinase inhibitors, and it can inhibit fibrinolysis. *SERPINE1* is closely related to the prognoses of head and neck squamous cell carcinoma (41), glioma (42), and gastric cancer (43). Finally, *DYDC2* is one of the marker genes of ciliated cells. The results of a single-cell study showed that there were secretory and ciliated tumor cells in endometrial and ovarian tumors, which were positively correlated with the disease-specific survival and OS of patients with endometrial carcinoma (44). This suggests that the *DYDC2* gene is related to the prognosis of tumors under certain conditions.

By studying colon cancer pathologic specimens, we confirmed that *DYDC2*, *MS4A15*, *MAGEA1*, *WNT7A*, *APOD*, and *SERPINE1* were highly expressed in colon cancer tissues. Our study indicated the potential of these six genes to promote cancer, and we validated the clinical prognostic value of the six-gene signature. GSEA results showed that the ECM-receptor interaction pathway, the TGF beta signaling pathway, etc. were positively correlated with risk score. In addition, the activity of Hedgehog signaling pathway, RNA degradation pathway, and other pathways decreased as the risk score increased, which suggested that the dysregulation of these pathways was closely related to tumor development. Therefore, the six-gene signature and enrichment analysis pathways screened and established in this study are worthy of further study to deepen our understanding of the mechanism of colon cancer occurrence and progression.

Many previous studies have tried to screen and construct prognostic marker models for colon cancer. For example, Zuo et al. created a six-gene signature based on the TCGA cohort (45). Sun et al. created an 11-gene signature based on the TCGA colon cancer cohort to guide COAD recurrence risk judgment (46). To further compare and confirm the advantages of signatures based on stem cell-related genes, the 2 above models were simultaneously analyzed. The results showed that our six-gene signature predicted prognosis better than the other two models. C-index analysis further showed that the overall performance of our model was better than that of the other two models. These results indicate that our model based on stem cell-related genes has a strong advantage and could be used by clinicians to help predict patient risk and provide guidance for patient evaluation and treatment.

Although this study was based on large sample multi-omics data, it still had some limitations. The conclusions in this study were mainly based on bioinformatics analysis, so further validation in *in vivo* and *in vitro* experiments is still needed. Second, some strong risk factors for colon cancer that might affect prognosis, such as diet and family history, were not included in this study. Finally, the samples in this research were all from retrospective studies, so it is necessary to conduct comprehensive and thorough research for the signature’s clinical application.

In summary, a six-gene signature, which showed satisfactory predictive performance in both training and validation cohorts, was constructed based on stem cell-related genes. The six genes, which were all independent prognostic factors, could be used for improved performance in the prediction of prognostic risk in colon cancer. Therefore, it is recommended to use this signature to assess the prognostic risk of patients with colon cancer.

## Data Availability Statement

Publicly available datasets were analyzed in this study, these can be found in The Cancer Genome Atlas (https://portal.gdc.cancer.gov/); the NCBI Gene Expression Omnibus (GSE39582, GSE17536).

## Ethics Statement

The studies involving human participants were reviewed and approved by the Research Ethics Committee of China Medical University and all patients signed informed consent forms to allow analyses to be performed on their tissue samples.

## Author Contributions

QS and XW contributed conception and design of the study. YL performed the experiment and statistical analysis. All authors contributed to the article and approved the submitted version.

## Conflict of Interest

The authors declare that the research was conducted in the absence of any commercial or financial relationships that could be construed as a potential conflict of interest.

## Supplementary Material

Supplementary Figure 1 Cophenetic, rss, and dispersion distribution with rank = 2–10, in which the cophenetic correlation is obtained based on the consistency matrix proposed by Brunet et al. It is used to reflect the stability of the cluster obtained from NMF. This value is between 0 and 1; the greater it is, the more stable between clusters. rss refers to residual sum of squares, which is used to reflect the clustering performance of the model. The smaller rss is, the better the model clustering effect. In theory, when each sample is clustered into a class, rss should be the smallest, but this condition is not actually available, so it needs to be combined with other indicators.

Supplementary Figure 2
**(A)** Sample distribution of three subtypes in different T stages; **(B)** Sample distribution of three subtypes in different N stage; **(C)** Sample distribution of three subtypes in different M stages; **(D)** Sample distribution of the three subtypes at different clinical stages; **(E)** Sample distribution of the three subtypes in males and females; **(F)** Sample distribution of the three subtypes at age >=60 and age <60.

Supplementary Figure 3
**(A)** B cell score between molecular subtypes; **(B)** CD4 cell score between molecular subtypes; **(C)** CD8 cell score between molecular subtypes; **(D)** Neutrophil cell score between molecular subtypes; **(E)** Macrophage cell score between molecular subtypes; **(F)** Dendritic cell score between molecular subtypes.

Supplementary Figure 4
**(A)** Volcano map of differentially expressed genes between C1/C2 subtypes; **(B)** volcano map of differentially expressed genes between C1/C3 subtypes; **(C)** intersection between two types of differential genes.

Supplementary Figure 5
**(A)** KEGG enrichment results of differentially expressed genes; **(B)** Reactome enrichment results of differentially expressed genes, where the color represents the significance of P value, the red represents the smaller P value, the blue represents the larger P value. The dot size represents the number of genes enriched into this pathway, the more the number, the larger the dot; **(C)** GO CC enrichment results; **(D)** GO MF enrichment results, where different colors represent different pathways, and the lines represent that genes and pathways are related.

Supplementary Figure 6 KM curves of the six genes. The abscissa represents survival time, and the ordinate represents survival rate.

## Abbreviations

COAD, Colon adenocarcinomas; TCGA, The Cancer Genome Atlas; NMF, Non-negative matrix factorization; PFS, progression free survival; FPKM, Fragments Per Kilobase Million; DFS, Disease-free survival; NMF, non-negative matrix clustering algorithm; FDR, false discovery rate; HR, hazard ratio; CI, Confidence interval; GSEA, Gene Set Enrichment Analysis.
