# Supplementary figures and images for: Identification and Validation of a Novel Six-Gene Prognostic Signature of Stem Cell Characteristic in Colon Cancer
Source: Front Oncol. 2021 Feb 19;10:571655. doi: 10.3389/fonc.2020.571655 (PMC7933554; doi:10.3389/fonc.2020.571655)

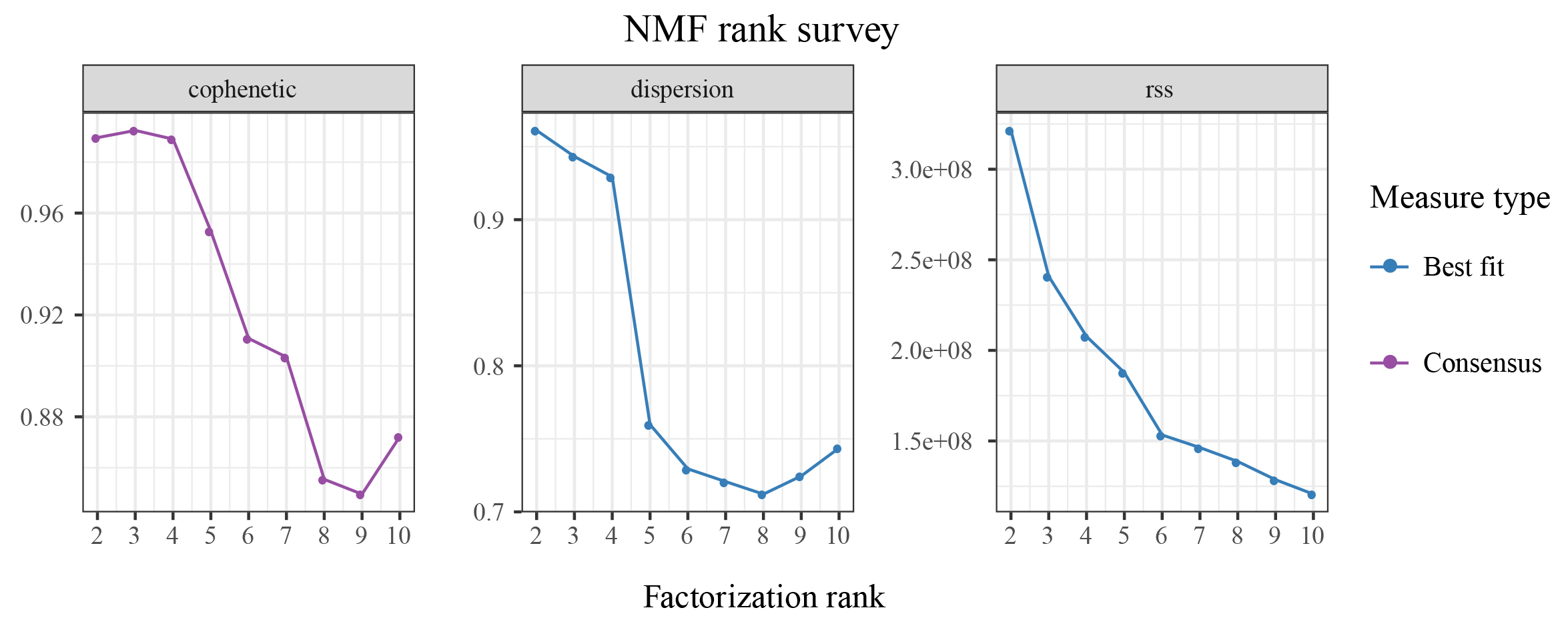

Supplement: Supplementary Figure 1 — Cophenetic, rss, and dispersion distribution with rank = 2–10, in which the cophenetic correlation is obtained based on the consistency matrix proposed by Brunet et al. It is used to reflect the stability of the cluster obtained from NMF. This value is between 0 and 1; the greater it is, the more stable between clusters. rss refers to residual sum of squares, which is used to reflect the clustering performance of the model. The smaller rss is, the better the model clustering effect. In theory, when each sample is clustered into a class, rss should be the smallest, but this condition is not actually available, so it needs to be combined with other indicators. [file Image_1.jpeg]

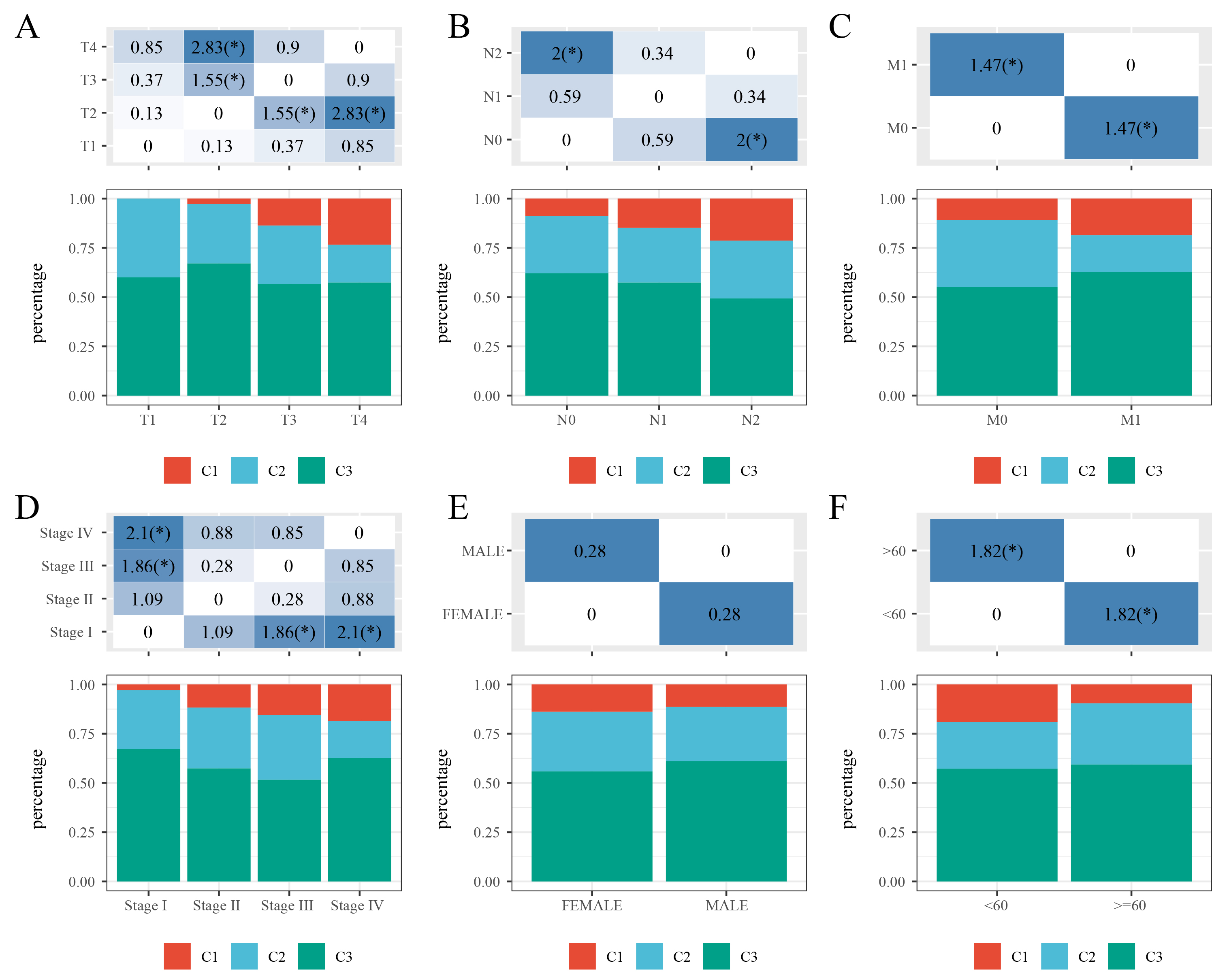

Supplement: Supplementary Figure 2 — (A) Sample distribution of three subtypes in different T stages; (B) Sample distribution of three subtypes in different N stage; (C) Sample distribution of three subtypes in different M stages; (D) Sample distribution of the three subtypes at different clinical stages; (E) Sample distribution of the three subtypes in males and females; (F) Sample distribution of the three subtypes at age >=60 and age <60. [file Image_2.jpeg]

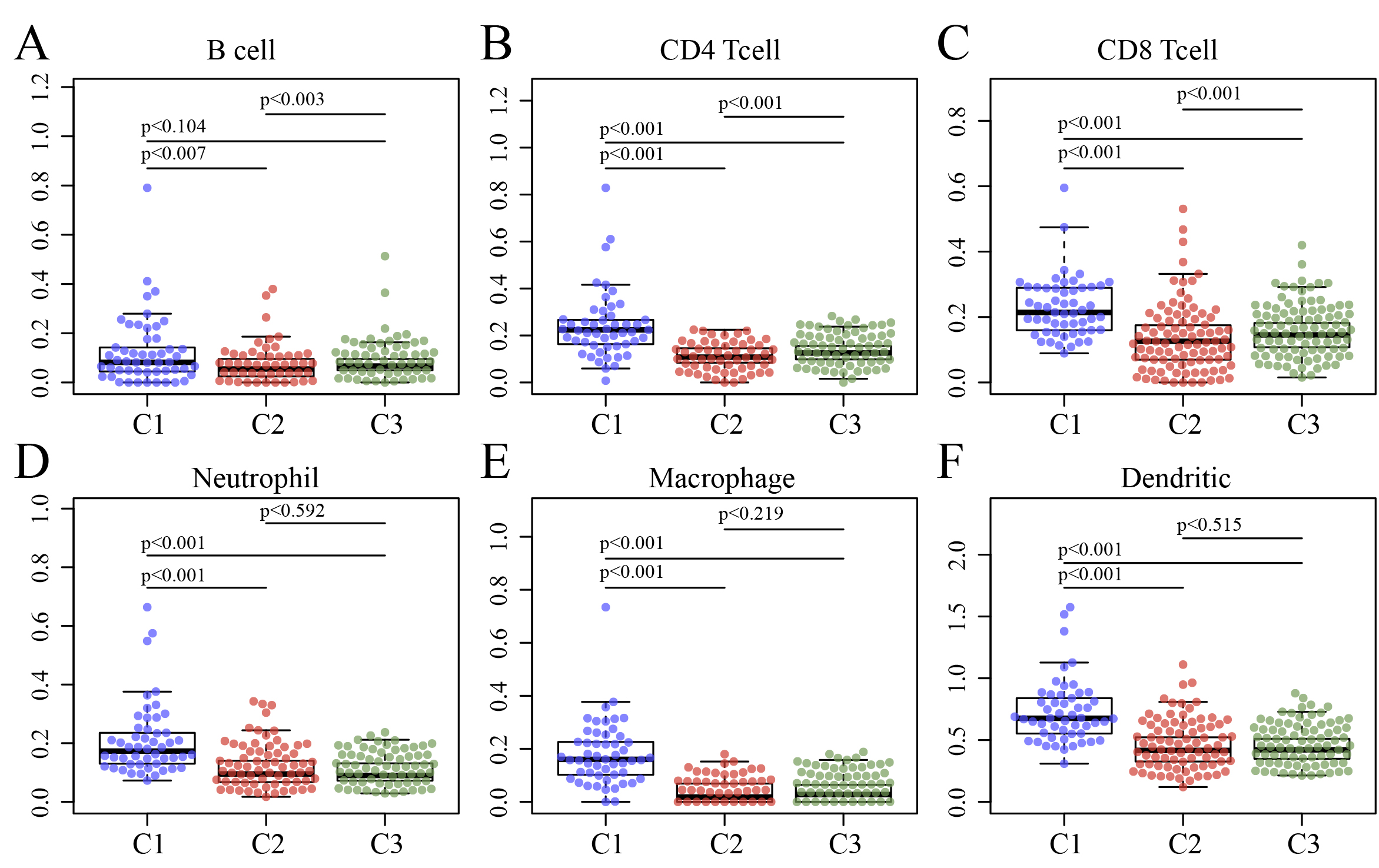

Supplement: Supplementary Figure 3 — (A) B cell score between molecular subtypes; (B) CD4 cell score between molecular subtypes; (C) CD8 cell score between molecular subtypes; (D) Neutrophil cell score between molecular subtypes; (E) Macrophage cell score between molecular subtypes; (F) Dendritic cell score between molecular subtypes. [file Image_3.jpeg]

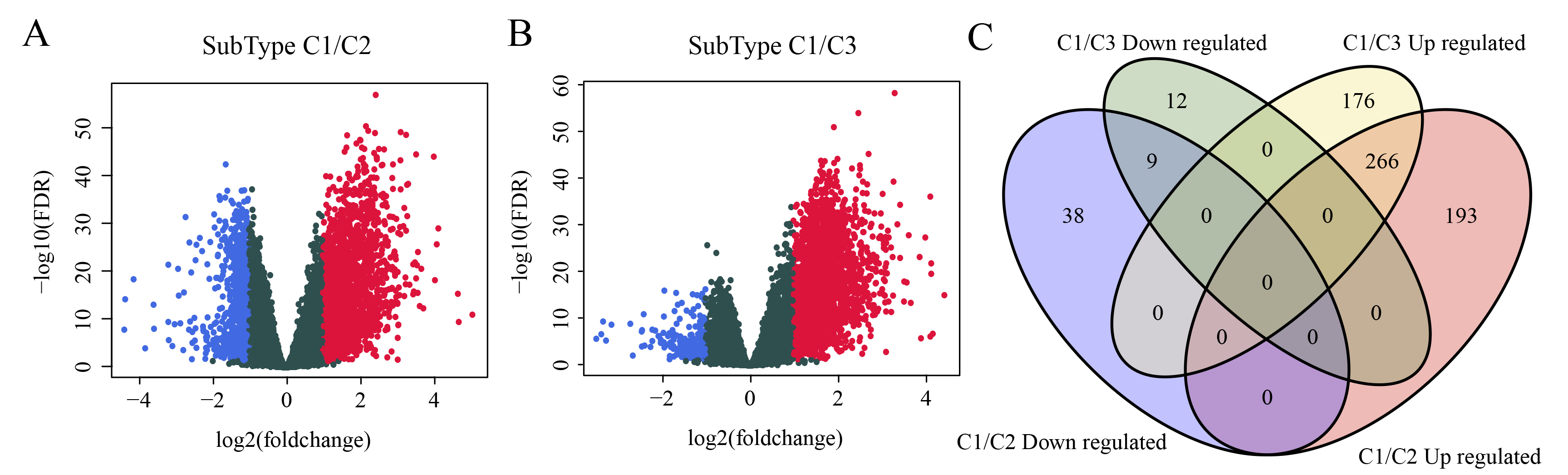

Supplement: Supplementary Figure 4 — (A) Volcano map of differentially expressed genes between C1/C2 subtypes; (B) volcano map of differentially expressed genes between C1/C3 subtypes; (C) intersection between two types of differential genes. [file Image_4.jpeg]

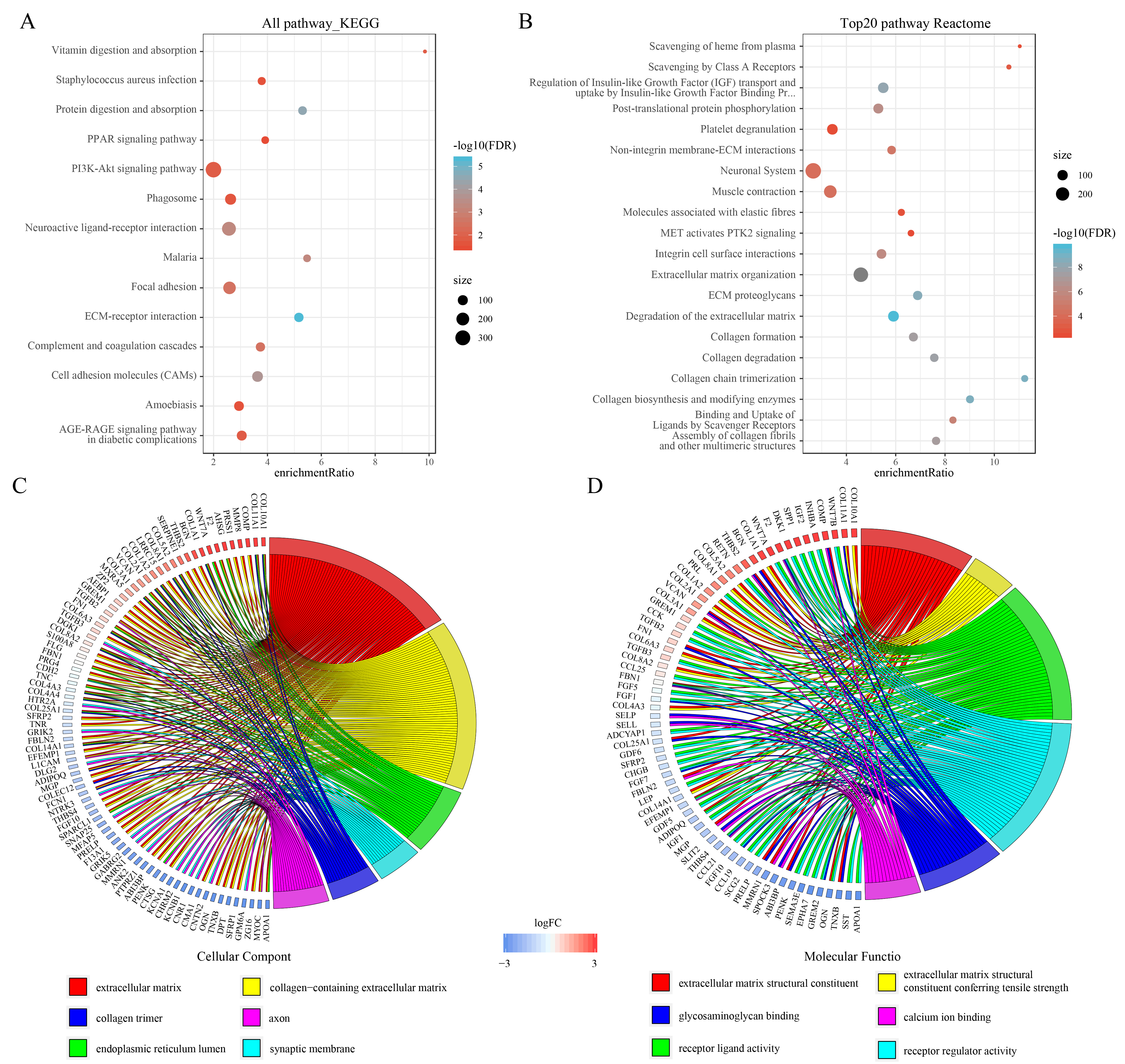

Supplement: Supplementary Figure 5 — (A) KEGG enrichment results of differentially expressed genes; (B) Reactome enrichment results of differentially expressed genes, where the color represents the significance of P value, the red represents the smaller P value, the blue represents the larger P value. The dot size represents the number of genes enriched into this pathway, the more the number, the larger the dot; (C) GO CC enrichment results; (D) GO MF enrichment results, where different colors represent different pathways, and the lines represent that genes and pathways are related. [file Image_5.jpeg]

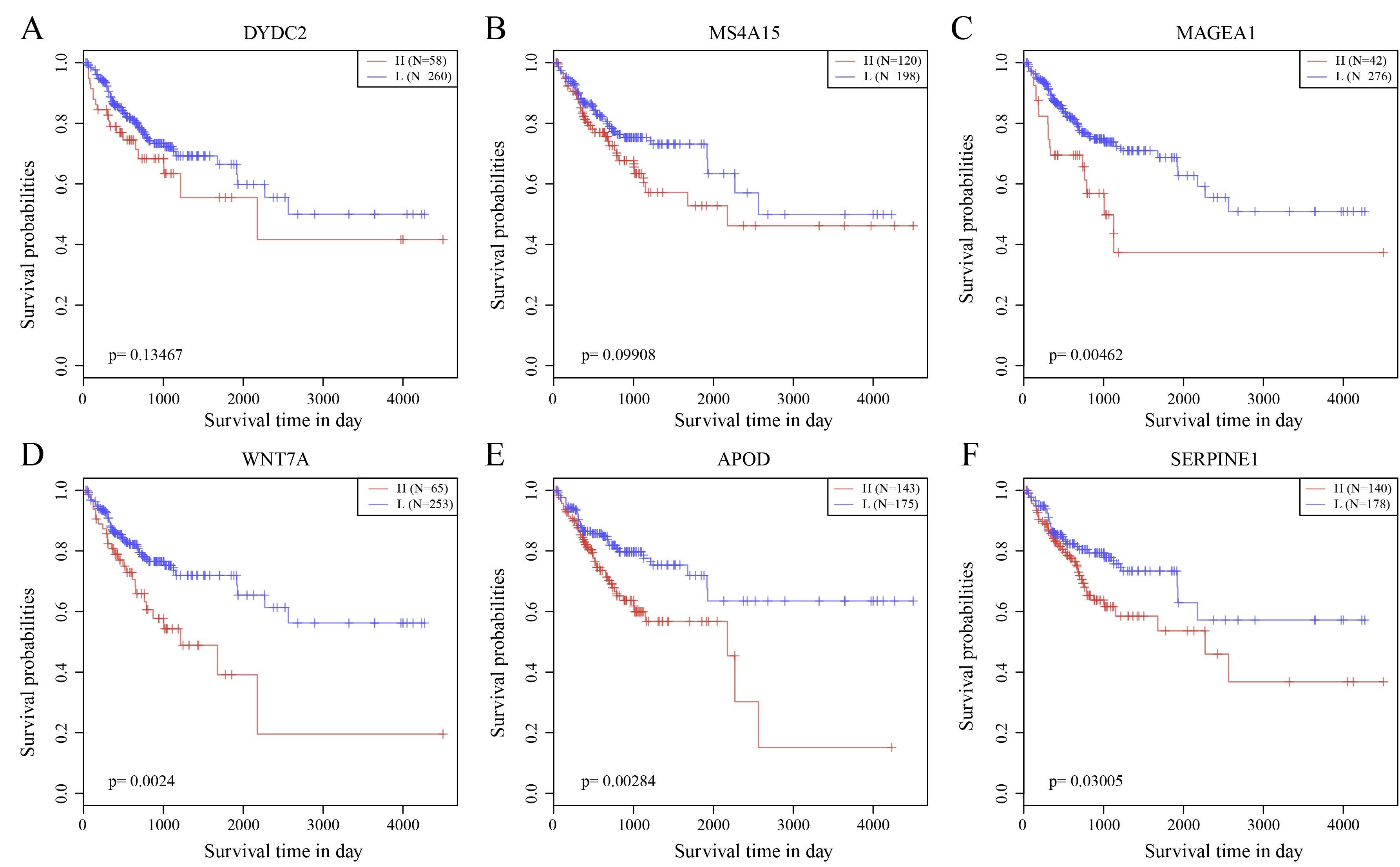

Supplement: Supplementary Figure 6 — KM curves of the six genes. The abscissa represents survival time, and the ordinate represents survival rate. [file Image_6.jpeg]
